# Anal canal cancer in Brazil: why should we pay more attention to the epidemiology of this rare disease?

**DOI:** 10.3332/ecancer.2020.1037

**Published:** 2020-05-07

**Authors:** Mauro DS Donadio, Rachel P Riechelmann

**Affiliations:** AC Camargo Cancer Center, São Paulo, 01509010, Brazil; a http://orcid.org/0000-0002-4705-4802; b http://orcid.org/0000-0002-0107-9617

**Keywords:** anal cancer, epidemiologic study, HPV vaccines, HIV raise, sexual behaviour

## Abstract

Anal canal cancer is one of the human papilloma virus (HPV)-associated diseases with increasing incidence. High-risk sexual behaviour and the resurgence of human immunodeficiency virus (HIV) infection, associated with low HPV vaccine coverage, are risk factors for the increased incidence of this cancer. In this paper, the authors point out pertinent questions regarding the greater exposure of the population to some risk factors and discuss the latest epidemiological data of these factors, particularly those of concern to emerging countries like Brazil. The authors also discuss policies adopted that have not been successful to combat the HIV and HPV rise and that have direct consequences on the incidence of anal canal cancer.

## Introduction and background

Squamous cell carcinoma of the anal canal, often broadly termed anal canal cancer, is a rare disease but its frequency is increasing, especially in high-risk groups [1]. Worldwide, age-standardised incidence and mortality rates for anal cancer are 0.53 and 0.20 (per 100,000), respectively, and the disease accounted for about 20,000 deaths in 2018 [2]. In emerging countries in Latin America, the incidence has been reported as 0.92%, but it is likely higher because national data are underestimated due to incomplete cancer registries [2, 3].

Known risk factors, except for age and immunosuppressive drug use, are behavioural [4]. Smoking significantly increases the risk of developing anal cancer, reaching a relative risk of 9.4 in men when compared to nonsmokers [5]. High-risk, multi-partner and anoreceptive sex also increase the relative risk of anal cancer [5]. A history of sexually transmitted diseases (STDs), such as herpes, gonorrhoea, syphilis, chlamydia and particularly the underlying human immunodeficiency virus (HIV) infection, is one of the most important risk factors [5]. Yet, the main risk factor is the human papilloma virus (HPV) infection [6, 7]. Increasing evidence indicates that oncogenic HPV strains, specifically subtypes 16 and 18, cause anal canal cancer, as it is known to occur in the cervix and head and neck [5]. Recent population-based studies by the US Centers for Disease Control (CDC) show that 91% of cervical, 75% of vaginal, 91% of anal and 70% of oropharyngeal neoplasms are attributable to any HPV type [8]. Each year in the United States, it is estimated that 44,000 new cancers are attributable to HPV [8].

Among the more than 240 types described, HPV 16 and 18 genotypes are considered of high risk because, by integrating their DNA into the host cell genome, they encode oncoproteins with stimulating properties, enabling the progression of preneoplastic lesions to invasive carcinoma [26]. The loss of E2 protein suppressor function after breakdown in its region during DNA integration results in increased expression of viral proteins E6, which inactivates protein p53 and E7 that inhibits protein Rb. The decreased activity of these cellular control proteins associated with increased expression of viral stimulating oncoproteins thus allows the immortalisation of infected cells and invasion, characterising carcinoma [9].

Currently, nearly 90% of anal cancer tumour samples are HPV positive, with different rates according to geographic location. HPV 16 infection is the most common, present in 86% of cases. In some cases, coinfection with multiple HPV types has been found [10].

An ongoing Brazilian study to determine the prevalence of HPV in sexually active women and men aged 16 to 25 years and to investigate regional differences in prevalence and virus types showed that for 5.812 women and 1.774 men analysed with median age of 20.6 years (95% CI 20.5–20.7), 17.7% of the study participants reported the presence of a previous STD or tested positive for the rapid HIV or syphilis tests. Regarding HPV, 35.2% (*n* = 2,669) of the collected samples have been tested for the presence of the virus and genotyped for subtype definition. Overall, the estimated prevalence of HPV was 54.6%, and high-risk HPV was present in 38.4% of participants. The study also showed that risky sexual behaviour was observed in 83.4% of respondents, with the average of sexual partners in the last year being 2.2 (95% CI 1.7–2.7), and the average of sexual partners in the last 5 years being 7.5 (95% CI 4.5–10.6) [11].

Because it is a strongly HPV-related disease, most (approximately 85%) of anal canal cancers are of squamous cell origin [6]. In the remaining cases, about 10% are adenocarcinomas and 5% are rare tumour types, such as melanoma, small cell carcinoma and metastatic tumours from other sites [5].

The modern treatment of this disease has been perfected over the years by many studies, such as that of Nigro *et al* [12] and is based on chemotherapy associated with radiotherapy, leaving surgery as a rescue option [13]. In the palliative scenario, carboplatin + paclitaxel has replaced cisplatin and 5FU as the first-line option following the randomised data from the interAACT trial, with an overall median survival gain (20 versus 12.3 months; hazard ratio (HR) 2.0; *p* = 0.014) and better tolerance profile [14]. In addition, pembrolizumab and nivolumab have shown some efficacy in initial immunotherapy studies, with response rates of 17% and 24%, respectively [15, 16]. One of the main ways to fight the disease, however, is based on the immunisation of the population against HPV, with the most commonly used agent being the quadrivalent vaccine [17, 18], while the evidence for anal oncotic cytology as a screening measure for high-risk groups is not robust enough for worldwide implementation [19].

Next, we will discuss some pertinent and worrying aspects on the increasing incidence of anal cancer in Brazil which may apply to other emerging countries.

## Discussion

### Tobacco use and reduction

Between 1989 and 2013, the percentage of adult smokers in Brazil dropped significantly due to the numerous actions taken by the National Tobacco Control Policy, granted in 1988 [20]. Official data show that in 1989, 34.8% of the population over 18 years was a smoker and that in the last survey of 2017–2018, this percentage dropped to 10.1% as shown in Table 1 [21]. Considering the period from 1989 to 2010, the drop in the percentage of smokers in Brazil was 46%, as a consequence of the implemented Tobacco Control Policies. It is estimated that around 420,000 deaths were prevented during this period [22], confirming the importance of public health policies that are well-designed and adherent by the population.

### Increased HIV incidence

Unfortunately, HIV/acquired immunodeficiency syndrome (AIDS) policies do not seem to achieve the same success. According to the HIV / AIDS epidemiological report of 2018, over the past 10 years, the number of people infected with HIV has increased exponentially (Table 1), with an increasing male predominance (male:female ratio has increased from 1.4 to 2.6), and the increased detection of AIDS occurs from 15 years of age for both sexes [23]. A change in the contagion pattern of the virus was detected, with decreased heterosexual transmission and increased among men who have sex with men (MSM). Male heterosexual sexual exposure fell from 44.3% to 34.9%, being stable among women (96.9% to 96.6%), and homosexual or bisexual increased from 49.1% to 62% [23].

However, the report also shows a decrease in the number of deaths associated with HIV [23], which contributes to the higher incidence of anal canal cancer attributed to two main factors: increased survival among individuals with HIV infection and increased high-risk sexual activity resulting in increased HPV acquisition rates and thus, higher risk of developing cancer over time. While HIV infection is a well-established risk factor for anal canal cancer, the associated risk of anal canal is not reduced by the use of highly active antiretroviral therapy (HAART) [5]. There is a 12-fold increased risk of anal cancer in people who have been infected with HIV for 15 years or more compared to those who have been infected with HIV for 5 years or less [5].

### Public immunisation policies

Another policy that has faced difficulties is immunisation against HPV. Two HPV vaccines (bivalent and quadrivalent) are licensed by the Food and Drug Administration (FDA) and available in Brazil. The quadrivalent HPV vaccine has been shown to prevent HPV 16 and HPV 18-associated vaginal, vulvar and anal precancer lesions and HPV 6 and HPV 11-associated anogenital warts [17, 18]. The 3-dose series has very high efficacy for the prevention of virus-associated cervical cancer [17].

Like cervical cancer, anal cancer is preceded by high-grade intraepithelial neoplasia [18]. Studies evaluating the effectiveness of immunisation showed that the rate of grade 2 or 3 anal intraepithelial neoplasia related to HPV 6, 11, 16 or 18 infection was reduced by 54.2% (95% CI 18, 0–75.3) in the intention-to-treat population and 74.9% (95% CI, 8.8–95.4) in the per-protocol efficacy population [18]. The corresponding risks of persistent anal infection with HPV 6, 11, 16 or 18 were reduced by 59.4% (95% CI, 43.0–71.4) and 94.9% (95% CI, 80.4–99.4), respectively, among MSM [18]. These data show that vaccines are effective prophylaxis. However, they do not prevent the progression of the existing infection to disease or treat the existing disease [24].

The American Advisory Committee on Immunisation Practices then recommends that girls and boys be routinely vaccinated at the age of 11 or 12 and, for those who were not vaccinated when they were younger, young women up to 26-year old and young men up to 21 years of age should be vaccinated. The committee also recommends that men who have sex with men be vaccinated by age 26 [25].

In Brazil, the Unified Health System included, in March 2014, the quadrivalent HPV vaccine in the National Immunisation Programme (NIP), currently available for girls aged 9 to 14 and boys aged 11 to 14, in two doses at 6 months intervals. For people living with HIV, transplanted and cancer patients undergoing chemotherapy and radiotherapy and who are 9 to 26 years, the quadrivalent HPV vaccine is available in three doses [26]. By June 2017, 18 million doses were applied to the female population nationwide, but only 7.1 million girls received the full two-dose vaccination schedule advocated by the Ministry of Health, which is 47% of the target audience. For boys, only 23.6% of the 3.61 million adolescents ages 12 to 13 received the first dose of the vaccine [26].

Low vaccination coverage, however, is not unique in emerging countries. In the United States, underuse of vaccines has been a permanent concern. A 2012 CDC report estimated that adolescent immunisation coverage was 54% for 1 dose and only 33% for the 3 dose recommendation. [27] Reasons cited for low penetrance include lack of knowledge about the benefits of the vaccine, concerns about the safety of the vaccine and parents’ perception that their children were not sexually active, and therefore did not need to be vaccinated [27].

Brazilian studies to ascertain the reasons for the low vaccination coverage showed that parents’ acceptance of the HPV vaccine for daughters and children under 18 was high (92% and 86%, respectively) [28]. Parents who accepted the HPV vaccine for their daughters but not for their boys were more likely to ignore that the vaccine is recommended for boys. Attitudes associated with acceptance of the HPV vaccine include: general belief in vaccines, trust in the NIP and effectiveness of the HPV vaccine. Less than a third of parents (30%) knew that there was a vaccine to prevent genital warts, and only 37% recognised that condoms are not fully protective against HPV infection. The most common reason for refusing HPV vaccination in both children was ‘fear of adverse reactions or events’ reported as the primary reason by 51% of parents. Among parents who refused HPV vaccination for boys (but accepted it for daughters), the most commonly reported reason was ‘HPV vaccine is not recommended for boys’ (74% as the main reason) [28].

However, it is known that 92.1% of vaccine-related adverse events are classified as non-serious. Among non-serious adverse events, the most commonly reported generalised symptoms were syncope, dizziness, nausea, headache, fever and hives; the most commonly reported local symptoms were injection site pain, redness and oedema. Among the 600,588 doses of quadrivalent vaccine given to women aged 9 to 26 years, there was no significant increased risk of any of the pre-specified adverse events following vaccination, including Guillain-Barré syndrome, seizures, syncope, appendicitis, stroke or venous thromboembolism, anaphylaxis and other allergic reactions [17].

Increased use of HPV vaccines by young adults may be the most cost-effective mechanism to prevent anal canal cancer morbidity and mortality in the coming decades. The major public health challenge is to develop accurate forms of communication about HPV for people to understand the importance of prevention and problems associated with the virus.

### HPV and HIV synergy

As we witness the exponential increase in the incidence of HIV infection, a new study shows that most young people exhibit high-risk sexual behaviour and a high prevalence of high-risk HPV [11]. People with HIV, even when effectively treated with HAART, have an increased risk and rate of HPV acquisition because they have B cell, T cell and NK cell dysfunction, persistent inflammation and persistent mucosal epithelial abnormalities. In addition, co-infected people have a higher frequency of multiple HPV types and an increased rate of HPV-related disease, including faster progression to malignancies [29].

Another previous Brazilian study showed that almost all women with HIV are infected with HPV, with high frequency of multiple HPV genotypes. Of the 208 HIV-infected women, 98% were found to be HPV DNA positive. Overall, reverse hybridisation genotyping revealed unique genotypes in 43 (21.1%) and multiple genotypes were detected in 161 (78.9%) of 204 HPV DNA positive patients, with an average of 3.1 genotypes per patient (ranging from 1 to 10 genotypes). HPV 6 was the most prevalent genotype and was observed in 39.2% of patients, followed by genotypes 51 (31.9%), 11 (26.0%), 18 (24.0%) and 16 (22.5%). Of the detected genotypes, 40.9% were low risk, 21.2% of the study population contained only high-risk genotypes, while 64.3% had high- and low-risk genotypes. The study also evaluated cervical oncotic cytology and found that at least one high-risk genotype was present in 89% of class I samples, 84% of class II samples and 82% of class III samples. Of the 28 patients with squamous intraepithelial lesions, 18% had exclusively low-risk genotypes, 46% had low- and high-risk genotypes and 36% had exclusively high-risk genotypes [30].

It seems logical, then, that the HIV-infected population should be prioritised in HPV immunisation campaigns. HIV, however, often reduces vaccine responsiveness and effectiveness. Even individuals who have been treated and who have suppressed viral loads for more than 5 years have specific defects in memory helper T-cell function, leading to reduced B-cell responses [29]. A randomised placebo-controlled clinical study to evaluate the efficacy of quadrivalent vaccine in the prevention of anal HPV in patients with HIV, early interrupted by futility, involved 575 HIV-positive individuals aged 41–52 years, median CD4 of 606 cells/μL, 88% with viral load <200 copies/mL. The primary endpoint was the efficacy of the vaccine against the new persistent HPV infection 6, 11, 16 and 18, which corresponded to 22%, considered non-significant. High baseline HPV seropositivity was observed, suggesting that vaccine efficacy may have been compromised by prevalent undetected subclinical / latent infections at baseline [31].

A Canadian cohort evaluated 432 HIV-positive patients aged 9–65 years (median CD4 of 500, 69% with viral load <50 copies/mL) who received three doses of quadrivalent vaccine. Of the women who met the inclusion criteria for the 2-year follow-up efficacy analyses (95.3% of those receiving 3 doses), the intention-to-treat population was 279 and the incidence rates were 2.3/100/year for persistent infection of genotypes 6, 11, 16 and 18 (most were due to HPV 18) and 2.3/100/year for genital warts. Persistent cases of HPV showed lower CD4 counts (median 333 cells/μL) [32]. In vaccinated women without HIV, the persistent infection rate is 0.1/100/year [29]. A third study on quadrivalent vaccine efficacy in patients who had been exposed or infected perinatally by HIV and subsequently vaccinated in adolescence showed that seroconversion to HPV 6, 11, 16 and 18 occurred in 83%, 84%, 90%, and 62% of those infected with HIV and 94%, 96%, 99% and 87% of those exposed to HIV, respectively (*p* < 0.05). There was a higher incidence of genital warts and abnormal cervical oncotic cytology in those infected with HIV [33].

There are several studies examining the immunogenicity of HPV vaccines in people with HIV, and in general, there is some reduction in HPV antibody levels compared to HIV negative individuals [29]. However, the lack of solid evidence of the effectiveness of the HPV vaccine in HIV is a significant concern and requires further clinical trials.

## Conclusion

Despite the decline in smoking, emerging countries like Brazil have witnessed a relentless upsurge in HIV infection, undoubtedly associated with a change in the now-at-risk population’s pattern of sexual behaviour. These important behavioural risk factors should cause the epidemiology of HPV infection, either by higher incidence or by co-infection with multiple subtypes and HIV, to change in the coming years causing an increased incidence of HPV-related tumours, among them anal canal carcinoma. There is still no solid evidence to screen anal cancer and HPV prophylaxis measures have faced serious difficulties with misinformation by the population, low coverage of immunisation programmes and likely lower efficacy of vaccines in immunosuppressed people. It is imperative and urgent to disseminate knowledge clearly and objectively about the risks related to high-risk sexual practices and to effectively guide the population’s protection measures, encouraging technical debate and ensuring access to health, insisting on improving immunisation coverage and HIV control programmes. Otherwise, in addition to witnessing an increase in other HPV-associated diseases, we will be outraged to see not only the rising incidence of anal canal cancer but also the cost it entails soar, whether related to its complex treatment or to quality of life and, of course, to life itself.

Since the increase in the use of HPV vaccines by young adults may be the most economical mechanism to prevent morbidity and mortality from cancer of the anal canal in the coming decades, a strategy that could increase vaccination coverage is the mandatory immunisation of all adolescents in schools. In addition to this, the expansion of public funds aimed at combating HIV / AIDS aimed at its prevention, that is, expanding the supply of condoms and carrying out wide-ranging advertisements emphasising the seriousness, costs and consequences of AIDS for the population and that an increase in this disease is closely linked to high-risk sexual behaviours, so it also suggests greater care in relation to these behaviours through educational materials in schools as well. High-risk individuals, such as MSM and HIV infected patients, should be routinely screened for anal cancer through clinical examination; the detection of early stage tumour improves the cure rates. The successful policy of combating tobacco must continue and count on the renewal of funds and the policies themselves. Finally, with the projected increase in cases of anal canal cancer, oncology services must be expanded and prepared with specialised professionals and infrastructure capable of meeting this demand.

## Conflicts of interest

The authors declare that they have no conflicts of interest.

## Funding source

This research did not receive any specific grant from funding agencies in the public, commercial or not-for-profit sectors.

## Warnings

The opinions expressed in the report presented are those of the authors and do not necessarily represent the official position of the institution to which they belong.

## Authors’ contributions

Both authors contributed equally for the manuscript.

## Financial support

None to declare.

## Figures and Tables

**Table 1.** Smoking and HIV frequencies and numbers from 2008 and 2018 in Brazil.

	2008	2018
Smoking*	18.5%	10.1%
HIV from high-risk sex**	49.1%	62%
HIV infection***	7.805	244.256

* Percentage of Brazilian population over 18 years.

** Reported cases of 13 years or older of HIV acquisition due to homosexual or bisexual relationship.

*** Total number of reported cases aged 13 and over.

Source: INCA—National Cancer Institute. National Tobacco Control Policy Observatory. 2019.

Ministry of Health. HIV / AIDS Epidemiological Report 2018
